# Phosphene Perception Relates to Visual Cortex Glutamate Levels and Covaries with Atypical Visuospatial Awareness

**DOI:** 10.1093/cercor/bhv015

**Published:** 2015-02-26

**Authors:** Devin B. Terhune, Elizabeth Murray, Jamie Near, Charlotte J. Stagg, Alan Cowey, Roi Cohen Kadosh

**Affiliations:** 1 Department of Experimental Psychology, Nuffield Department of Clinical Neurosciences, University of Oxford, Oxford, UK; 2 Centre for Functional MRI of the Brain (FMRIB), Nuffield Department of Clinical Neurosciences, University of Oxford, Oxford, UK; 3 Douglas Mental Health University Institute and Department of Psychiatry, McGill University, Montreal, Canada

**Keywords:** awareness, GABA, glutamate, phosphene, synesthesia, TMS, visual perception

## Abstract

Phosphenes are illusory visual percepts produced by the application of transcranial magnetic stimulation to occipital cortex. Phosphene thresholds, the minimum stimulation intensity required to reliably produce phosphenes, are widely used as an index of cortical excitability. However, the neural basis of phosphene thresholds and their relationship to individual differences in visual cognition are poorly understood. Here, we investigated the neurochemical basis of phosphene perception by measuring basal GABA and glutamate levels in primary visual cortex using magnetic resonance spectroscopy. We further examined whether phosphene thresholds would relate to the visuospatial phenomenology of grapheme-color synesthesia, a condition characterized by atypical binding and involuntary color photisms. Phosphene thresholds negatively correlated with glutamate concentrations in visual cortex, with lower thresholds associated with elevated glutamate. This relationship was robust, present in both controls and synesthetes, and exhibited neurochemical, topographic, and threshold specificity. Projector synesthetes, who experience color photisms as spatially colocalized with inducing graphemes, displayed lower phosphene thresholds than associator synesthetes, who experience photisms as internal images, with both exhibiting lower thresholds than controls. These results suggest that phosphene perception is driven by interindividual variation in glutamatergic activity in primary visual cortex and relates to cortical processes underlying individual differences in visuospatial awareness.

## Introduction

The application of transcranial magnetic stimulation (TMS) to visual cortex reliably produces illusory visual percepts known as “phosphenes” (Cowey and Walsh 2000). These percepts are characterized by flashes of light and vary in phenomenology depending on stimulation parameters (Cowey and Walsh 2000; Salminen-Vaparanta et al. 2014). Phosphene thresholds, the minimum stimulation intensity required to reliably elicit phosphenes, are stable within individuals (Stewart et al. 2001; Boroojerdi et al. 2002) and widely used as an index of visual cortex integrity or excitability (Cowey and Walsh 2000; Stewart et al. 2001; Bestmann et al. 2007). They may also be valuable in elucidating the role of visual cortex in visual awareness (Sparing et al. 2002; Salminen-Vaparanta et al. 2014) and the mechanisms underlying visual cortex plasticity (Boroojerdi et al. 2001). However, their neurochemical basis and relationship to individual differences in visual cognition are poorly understood.

Different lines of indirect evidence suggest that phosphene thresholds may be driven by variability in glutamate or GABA levels in primary visual cortex. Motor cortex glutamate levels have been shown to correlate with physiological measures of motor cortex excitability (Stagg et al. 2011), suggesting a similar relationship may hold between phosphene thresholds and glutamate levels in primary visual cortex. On the other hand, it has been suggested that the increase of phosphene thresholds following the intake of anticonvulsants (Mulleners et al. 2002) may occur through an upregulation of GABA tone (e.g., Brigo, Bongiovanni, et al. 2013), suggesting a possible relation between phosphene thresholds and local GABA levels. Finally, anodal transcranial direct current stimulation, which depolarizes local resting membrane potentials, has been shown to lower phosphene thresholds (Antal et al. 2003), although it is not yet clear whether this is caused by a reduction in GABA (Stagg et al. 2009) or an enhancement of glutamate (Siniatchkin et al. 2012).

Despite the widespread use of phosphene thresholds as an index of occipital cortex excitability, their relation to visual cognition is poorly understood. One strand of evidence for the functional significance of phosphenes comes from the results of Bestmann et al. (2007), who showed that top-down spatial attention selectively enhances cortical excitability to facilitate visual awareness. This suggests that phosphene thresholds may reflect cortical processes underlying variability in visuospatial awareness. Grapheme-color synesthesia, a neurological condition characterized by atypical binding and visual cortex hyperexcitability (Terhune et al. 2011), in which letters and numerals involuntarily elicit color photisms (Rouw and Scholte 2010), provides a valuable model of individual differences in visuospatial awareness. Most synesthetes experience color photisms as visual images (“associators”), whereas a small subset experiences photisms as spatially colocalized with the inducing grapheme (“projectors”) (Dixon et al. 2004). These visuospatial phenomenological differences have been proposed to involve the recruitment of different spatial reference frames during photism perception (Ward et al. 2007) and appear to be related to differential functioning in primary visual cortex (Rouw and Scholte 2010). If phosphene thresholds index cortical processes underlying individual differences in visuospatial awareness, we would expect projectors to display lower phosphene thresholds than associators.

Here, we assessed whether TMS phosphene thresholds are related to basal concentrations of GABA and glutamate in primary visual cortex, as measured by magnetic resonance spectroscopy (MRS). We further tested the prediction that phosphene thresholds would covary with individual differences in the visuospatial phenomenology of synesthesia.

## Methods

### Participants

Eleven nonsynesthetes (8 female, *M*_Age_ ± SE = 23.1 ± 1.6) and 10 grapheme-color synesthetes (7 female, 22.3 ± 1.1, 7 associators; 3 projectors), all right-handed, participated in the TMS study; all but one synesthete took part in the MRS study. None of the controls or synesthetes were in our previous study of cortical excitability in synesthesia (Terhune et al. 2011). TMS and MRS sessions were done on separate days. Participants provided informed consent in accordance with approval from a local ethics committee. Participants did not have a personal or family history of epilepsy, fainting, migraines, metallic implants, or serious mental or neurological illness, and none were currently using noncontraceptive medication.

### Synesthesia Consistency and Phenomenology

Controls and synesthetes identified color associations for the digits 0–9 and were administered a structured interview on 2 separate occasions (controls: 35 ± 14 days; synesthetes: 60 ± 13, unequal variance *t* = 1.3, *P* = 0.20). The mean Euclidean color distance between colors for the digits at the 2 time points was used as a measure of consistency (Rothen et al. 2013). Synesthetes displayed lower values, 17.9 ± 2.5, reflecting greater consistency of grapheme-color associations, than controls, 103.5 ± 8.0, unequal variance *t* = 10.21, *P* < 0.001, including when controlling for the number of days between sessions, *F*_1,18_ = 108.22, *P* < 0.001, *η*_p_^2^ = 0.86.

Visuospatial phenomenology was measured in 2 ways. First, synesthetes were presented with achromatic graphemes against a gray background and were asked about the visuospatial location of their color photisms, namely whether the photisms were perceived to be spatially proximal to the inducing stimulus, in space between the stimulus and the individual, or as visual images. They were classified as projectors if they reported experiencing color photisms as spatially colocalized with the inducing grapheme and as associators if they reported that photisms were experienced as mental images (Dixon et al. 2004; Ward et al. 2007). None of the participants reported photisms that were spatially localized between the stimulus and the percipient (Ward et al. 2007; van Leeuwen et al. 2011). Second, following previous studies that used questionnaires to provide a continuous measure of individual differences among synesthetes (Rouw and Scholte 2007; Skelton et al. 2009; Rouw and Scholte 2010), participants completed the “Illustrated Synaesthetic Experience Questionnaire” (ISEQ; Skelton et al. 2009), a self-report measure that measures the phenomenology, including visuospatial location, of synesthetic color photisms. The ISEQ displayed acceptable internal consistency in the sample of synesthetes (Cronbach's *α* = .73). Projectors (*M* = 3.0, SE = 0.42) displayed larger values on the associator–projector difference score (higher values indicate greater projector-type phenomenology) than associators (*M* = −3.74, SE = 0.49), *t*(8) = 8.29, *P* < 0.001. In addition, all synesthetes were correctly classified as projector or associator in accordance with the stimulus-based assessment above with 0 “undetermined” classifications, according to the ISEQ associator–projector cutoff criteria for the scale (Skelton et al. 2009), thereby corroborating the stimulus-based assessment.

### Transcranial Magnetic Stimulation

An experimenter blind to group measured stimulation thresholds using a Magstim TM model (Magstim) via a 70-mm figure-of-eight coil using three-pulse trains with interpulse intervals of 100 ms. Participants wore a lycra swimming cap to mark the optimal positions for the coil placement for determining phosphene thresholds and motor thresholds, included as a control. The participant's head was supported by a chin rest, and the TMS coil was manually held against the stimulation site by the experimenter on each trial to ensure precise coil position as the use of a clamp may reduce stimulation site precision because of head movements produced by verbal responses (Abrahamyan et al. 2011). Stimulation sites (motor or visual cortices) were initially determined by stimulating multiple sites 6 times (or more, as required) at 50% stimulation intensity within 1 cm of the measured location with the stimulation sites being selected as those that were associated with the strongest, most reliable, and most precise motor and phosphene responses. Motor thresholds were measured using the observation of movement method, which is known to be highly reliable (Varnava et al. 2011). This method was used instead of motor-evoked potentials because it relies on a subjective judgment by the participant and thus provides a better control measure for phosphene thresholds. Motor thresholds were measured by placing the coil tangential to the scalp, with the handle pointing 45° postero-laterally while participants pressed together the index finger and thumb of the right hand and sat with eyes open (to observe any movements). Left motor cortex (5 cm lateral and left of the vertex and 2 cm rostral of the lateral site) was stimulated first at 50% stimulation intensity and ramped up or down as necessary to identify a site that reliably produced twitches in the interodosseus muscle of the right hand. After each pulse train, the participant and experimenter judged whether a movement was made and adjusted the stimulation intensity (initial intensity was 50%) using a modified binary search algorithm (Tyrrell and Owens 1988). Phosphene thresholds were measured following a period of eyes-closed dark adaptation. The TMS coil was placed with the handle in the horizontal position and the center of the confluence of the 2 coils on the midline of the skull, 2 cm dorsal of the inion, corresponding approximately to the representation of the fovea-macula in V1. Participants reported whether they experienced a phosphene after each stimulation with intensity subsequently adjusted as mentioned earlier. Vertex was stimulated 10 times at the phosphene threshold intensity to control for nonspecific (somatosensory or acoustic) effects of TMS, and participants (1 control, 1 synesthete) were excluded if they reported phosphenes on 5 or more trials.

## Magnetic Resonance Spectroscopy

### MRI Data Acquisition

**Figure 1.**
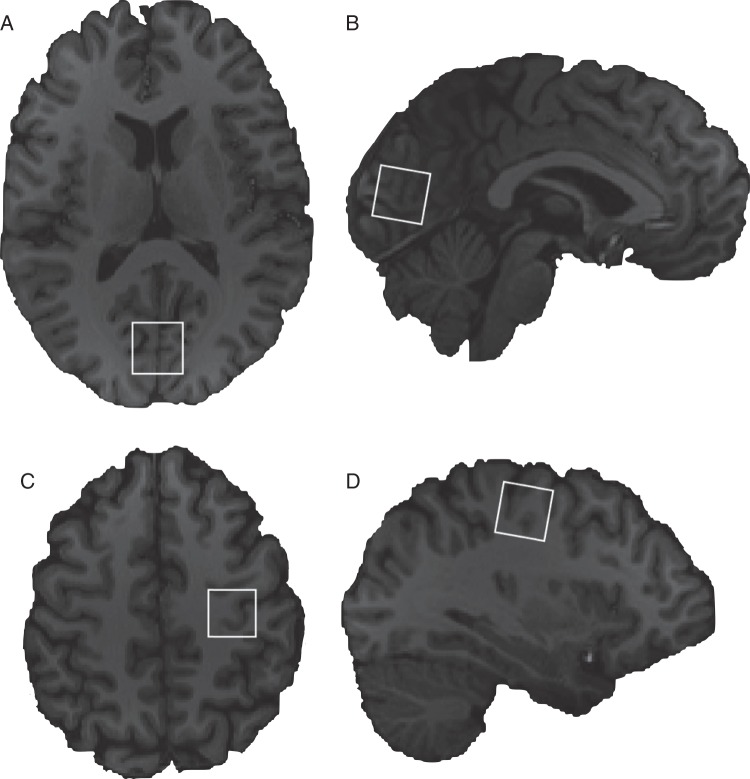
MRS voxel locations from a randomly selected participant. Shown are visual cortex axial (*A*) and saggital (*B*) views and left motor cortex axial (*C*) and saggital (*D*) views. Images are presented according to radiological convention.

The MRS data from the controls in this study were reported in a previous paper (Terhune, Russo, et al. 2014). Participants were scanned on a 3T Siemens scanner (Erlangen) with a body coil transmitter and a 32-channel receive head array. We first acquired a high-resolution T1-weighted scan using an MPRAGE (magnetization-prepared rapid gradient echo) sequence (Stagg et al. 2011). Short-TE MRS data were next acquired in two 2 × 2 × 2 cm voxels localized in primary visual cortex and primary motor cortex in the left hemisphere (the hand knob area of the middle central culcus; Yousry et al. 1997; Sastre-Janer et al. 1998) (Fig. 1) under eyes-open conditions in counterbalanced order. Shimming was performed using the vendor-provided automated shim tool. Short-TE MR spectra were acquired with the SPECIAL (spin-echo full-intensity acquired localized) sequence (2048 Points, spectral width = 2000Hz, TR/TE = 4000/8.5 ms, 128 Averages) (Mekle et al. 2009). Outer volume suppression was applied prior to each scan to saturate spins on all 6 sides of the voxel of interest, and VAPOR (variable power RF pulses with optimized relaxation delays) water suppression was used (Tkáč et al. 2001). Lastly, 8 averages of water-unsuppressed data were acquired with the same localization scheme.

### MRS Postprocessing and Analysis

Initial postprocessing was performed using in-house MATLAB-based (Natick) software, as previously described (Near et al. 2013). 32-channel data were recombined in a weighted fashion, with coil weights and phases determined using the magnitude and phase, respectively, of the first time-domain point of the water-unsuppressed data. Next, the subspectra resulting from SPECIAL preinversion on/off scans were subtracted from each other. Following subtraction, motion-corrupted scans were identified by a “deviation metric” for each individual scan (subtracting the scan from the average of all scans and then computing the root-mean-square of all of the spectral points in the difference vector). Scans whose metrics fell more than 2.6 standard deviations above the average were deemed to have been corrupted by motion and other factors and were removed, and this procedure was repeated until no motion-corrupted scans remained. Next, a frequency and phase drift correction was performed. This was achieved by least-squares fitting of each scan to the first scan in the series, using frequency and phase as adjustment parameters. This procedure was performed in the time domain, using only the first 40 ms of data. Following frequency and phase alignment of the scans, signal averaging was performed, resulting in a fully processed short-TE spectrum. All MRS data were analyzed in LCModel (Provencher 2001) using a simulated basis set that consisted of 22 individual metabolite signals. Line width (full width half maximum) was below 0.065 ppm (∼8 Hz) for all motor and visual cortex data, and there were no signal-to-noise ratio outliers (Carling 2000). Raw GABA and glutamate values were normalized by referencing to creatine, as is typically done (Ramadan et al. 2013) (these ratios are henceforth referred to as “concentrations”). MRS-derived neurochemical concentrations have been shown to be consistent over short and long periods of time (O'Gorman et al. 2011; Near et al. 2014). T1-weighted anatomical scans were segmented into gray and white matter using FAST (FMRIB's automated segmentation tool) (Smith 2002) in order to compute the percentage of gray and white matter in each voxel for control analyses.

### Statistical Analyses

Data were analyzed using MATLAB. Correlational data were non-normally distributed, and thus Spearman correlation coefficients were computed for all analyses. Participant group was partialled out in all analyses relating neurochemicals and stimulation thresholds except in the case of correlations that were computed separately in each group. There was a single bivariate outlier in 3 data pairs: visual cortex glutamate × GABA; visual × motor cortex GABA; and visual cortex glutamate × motor thresholds, identified using an adjusted boxplot rule (Carling 2000) and removed in the computation of skipped correlations (Wilcox 2004). We computed 95% confidence intervals (CIs) for different statistics (correlation coefficients, effect sizes, and means) using the bias-corrected and accelerated percentile bootstrap method (10 000 samples) (Efron 1987). Correlations were contrasted by bootstrap resampling data pairs, re-computing the coefficient difference, and then calculating the CIs of this distribution. We contrasted stimulation thresholds and neurochemicals across groups using mixed-model ANOVAs. Subsidiary analyses used planned comparisons and post hoc Tukey HSD tests.

## Results

### Relationships among Stimulation Thresholds and Neurochemicals

Motor and phosphene thresholds were uncorrelated in the total sample, *r*_s_ = −0.13, *P* = 0.62 (CIs: −0.58, 0.45), including when controlling for Group, *r*_s_ = −0.19, *P* = 0.47 (CIs: −0.71, 0.40). GABA and glutamate concentrations correlated in motor, *r*_s_ = .81, *P* < 0.001 (CIs: 0.50, 0.96), but not visual, *r*_s_ = .36, *P* = 0.15 (CIs: −0.24, 0.72), cortex. GABA concentrations across regions did not correlate, *r*_s_ = −0.41, *P* = 0.12 (CIs: −0.79, 0.23), nor did glutamate concentrations, *r*_s_ = −0.36, *P* = 0.18 (CIs: −0.76, 0.28).

### Phosphene Thresholds Selectively Predict Visual Cortex Glutamate Concentrations

**Figure 2.**
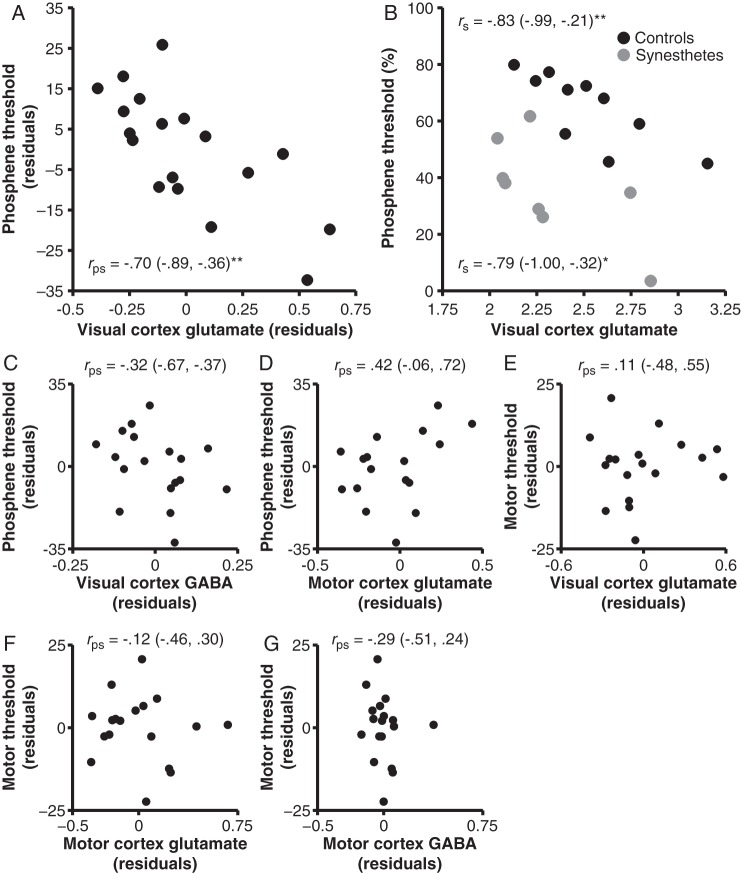
Relationships between stimulation thresholds and neurochemical concentrations. (*A*,*B*) Phosphene thresholds negatively correlated with visual cortex glutamate concentrations in the total sample (*A*) and controls and synesthetes independently (*B*). Phosphene thresholds did not correlate with visual cortex GABA (*C*) or motor cortex glutamate concentrations (*D*) and motor thresholds did not correlate with visual cortex glutamate concentrations (*E*), motor cortex cortex glutamate concentrations (*F*), or motor cortex GABA concentrations (*G*). (*A,C–E*) Data reflect residuals (controlling for Group). Bracketed values indicate bootstrap 95% confidence intervals. *P < 0.05, **P < 0.01.

Our primary set of analyses contrasted the predictions that phosphene thresholds would be negatively associated with glutamate concentrations or positively associated with GABA concentrations. In support of the hypothesis that variation in phosphene thresholds is driven by interindividual differences in occipital glutamate levels, visual cortex glutamate concentrations correlated strongly and negatively with phosphene thresholds (Fig. 2*A,B*). Lower thresholds were associated with greater glutamate in the total sample (controlling for group; Fig. 2*A*) and in both controls and synesthetes independently (Fig. 2*B*). This relationship remained significant when controlling for gray and white matter percentages within the visual cortex voxel, *r*_ps_ = −0.70, *P* = 0.004 (CIs: −0.91, −0.24), and visual cortex GABA concentrations, *r*_ps_ = −0.67, *P* = 0.005 (CIs: −0.88, −0.22). In contrast, visual cortex GABA concentrations were unrelated to phosphene thresholds, *P* = 0.21 (Fig. 2*C*), including when visual cortex glutamate concentrations were partialled out, *r*_ps_ = −0.11, *P* = 0.70 (CIs: −0.66, 0.48). Moreover, phosphene thresholds correlated more strongly with visual cortex glutamate than GABA concentrations (median difference: −0.37; CIs: −1.02, −0.02). These analyses demonstrate that this relationship is present in both controls and synesthetes and is independent of morphometric differences in the voxels as well as local GABA concentrations. We next undertook 2 series of control analyses to further clarify the topographic and threshold specificity of this relationship.

We first assessed whether the glutamate–phosphene correlation was specific to visual cortex and not due to an association between phosphene thresholds and general cortical glutamate concentrations. Phosphene thresholds correlated with visual cortex glutamate concentrations when partialling out motor cortex glutamate concentrations, *r*_ps_ = −0.68, *P* = 0.004 (CIs: −0.90, −0.28). In contrast, phosphene thresholds were unrelated to motor cortex glutamate concentrations, *P* = 0.09 (Fig. 2*D*), including when controlling for visual cortex glutamate concentrations, *r*_ps_ = .35, *P* = 0.18 (CIs: −0.18, 0.70). Furthermore, phosphene thresholds correlated more strongly with visual than motor cortex glutamate concentrations (median difference: −1.11, CIs: −1.51, −0.64). These analyses demonstrate the topographic specificity of the association between phosphene thresholds and visual cortex glutamate concentrations.

Our second set of control analyses examined whether the glutamate–phosphene relationship was specific to phosphene thresholds and not an artifact of a general relationship between visual cortex glutamate levels and global cortical excitability. This relationship remained stable when controlling for motor thresholds, *r*_ps_ = −0.70, *P* = 0.003 (CIs: −0.90, −0.27), whereas motor thresholds were unrelated to visual cortex glutamate concentrations, *P* = 0.68 (Fig. 2*E*), including when controlling for phosphene thresholds, *r*_ps_ = −0.03, *P* = 0.90 (CIs: −0.66, 0.46). In addition, visual cortex glutamate concentrations correlated more strongly with phosphene than motor thresholds (median difference: −0.81, CIs: −1.32, −0.21). These analyses indicate that the observed glutamate–phosphene relationship exhibits threshold specificity.

We next conducted a series of exploratory analyses investigating the neurochemical correlates of TMS motor thresholds. Motor thresholds were unrelated to motor cortex glutamate, *P* = 0.66 (Fig. 2*F*), or GABA, *P* = 0.27 (Fig. 2*G*), concentrations. The magnitude of these coefficients was unaltered when group was omitted as a covariate. These analyses suggest that motor thresholds, as assessed by the observation of movement method, are unrelated to motor cortex neurochemical concentrations.

### Phosphene Thresholds Predict Synesthesia Phenomenology

**Figure 3.**
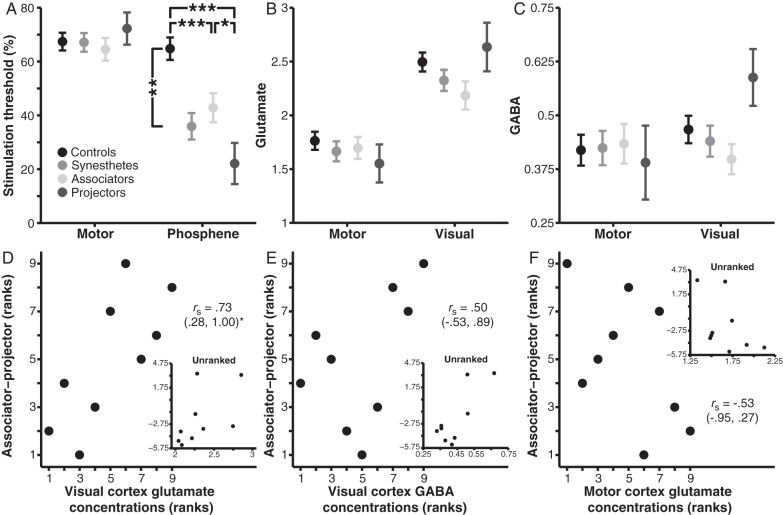
Stimulation thresholds and neurochemical concentrations in controls and synesthetes. Phosphene thresholds, but not motor thresholds, were lower in synesthetes than controls and varied with synesthesia phenomenology (*A*). (*B*,*C*) Neurochemical concentrations did not differ across groups in Glutamate (*B*) or GABA (*C*). (*D–F*) Ranked data relating ISEQ associator–projector scores (higher values reflect projector-type phenomenology) (unranked data are presented in insets). Associator–projector scores positively correlated with visual cortex glutamate concentrations (*D*), but not visual cortex GABA concentrations (*E*) or motor cortex glutamate concentrations (*F*). Error bars represent one standard error. Bracketed values indicate bootstrap 95% confidence intervals. *P < 0.05, **P < 0.01, ***P < 0.001.

Next we contrasted motor and phosphene thresholds in controls and synesthetes to investigate the effect of synesthesia on visual cortex excitability. A 2 × 2 mixed-model ANOVA on stimulation thresholds revealed main effects of Stimulation region (motor vs. visual cortex), *F*_1,17_ = 13.99, *P* = 0.002, *η*_p_^2^ = 0.45 (CIs: 0.09, 0.66), and Group (controls vs. synesthetes), *F*_1,17_ = 15.54, *P* = 0.001, *η*_p_^2^ = 0.48 (CIs: 0.11, 0.67), which were moderated by a Region × Group interaction, *F*_1,17_ = 9.97, *P* = 0.006, *η*_p_^2^ = 0.37 (CIs: 0.09, 0.72) (Fig. 3*A*). Subsidiary analyses showed that synesthetes exhibited lower phosphene thresholds than controls, *F*_1,17_ = 18.29, *P* = 0.001, *η*^2^ = .52 (CIs: 0.27, 0.79), whereas the 2 groups did not differ in motor thresholds, *F*_1,17_ < 0.01, *P* = 0.95, *η*^2^ < 0.01 (CIs: 0.00, 0.28), thus replicating previous work (Terhune et al. 2011).

A second ANOVA sought to elucidate the relationship between phosphene thresholds and individual differences in visuospatial awareness by contrasting stimulation thresholds in controls, associators, and projectors (Fig 3*A*). This analysis revealed main effects of Stimulation region, *F*_1,16_ = 29.81, *P* < 0.001, *η*_p_^2^ = 0.65 (CIs: 0.29, 0.79) and Group, *F*_2,16_ = 8.59, *P* = 0.003, *η*_p_^2^ = 0.52 (CIs: 0.10, 0.69), which were qualified by a Region × Group interaction, *F*_2,16_ = 8.84, *P* = 0.003, *η*_p_^2^ = 0.52 (CIs: 0.33, 0.80). As predicted, subsidiary analyses revealed a main effect of Group on phosphene thresholds, *F*_2,16_ = 13.70, *P* < 0.001, *η*_p_^2^ = 0.63 (CIs: 0.25, 0.81), with projectors displaying lower phosphene thresholds than associators, *P* < 0.05, *η*^2^ = .39 (CIs: 0.07, 0.82), both of whom exhibited lower thresholds than controls, *P* < 0.001, *η*^2^ = .67 (CIs: 0.49, 0.90), *P* < 0.001, *η*^2^ = .45 (CIs: 0.14, 0.79), respectively. In contrast, the 3 groups did not differ in motor thresholds, *F*_2,16_ = 0.56, *P* = 0.58, *η*_p_^2^ = 0.07 (CIs: 0.00, 0.22). Given the sample sizes of the 2 synesthesia subtypes, we sought to replicate these results using nonparametric bootstrap resampling to compute the 95% CIs for each mean. These analyses showed that the 3 groups had nonoverlapping distributions for phosphene thresholds (controls [*M* = 64.8, CIs: 56.6, 71.7], associators [*M* = 42.8, CIs: 35.0, 53.6], projectors [*M* = 22.1, CIs: 3.5, 33.2]), but overlapping distributions for motor thresholds (controls [*M* = 67.4, CIs: 62.5, 72.1], associators [*M* = 64.5, CIs: 54.1, 75.1], projectors [*M* = 72.3, CIs: 70.1, 76.1]), and thereby corroborated the parametric analyses.

We further investigated the association between synesthesia phenomenology and visual cortex excitability by using the ISEQ associator–projector difference score, which provides a continuous measure of visuospatial phenomenology of synesthetic color photisms (higher values indicate greater projector-type phenomenology). ISEQ scores were negatively correlated with phosphene thresholds, *r*_s_ = −0.81, *P* = 0.02 (CIs: −1.0, −0.10), which is consistent with lower thresholds being associated with projector-type phenomenology. This relationship remained significant when controlling for motor thresholds, *r*_ps_ = −0.88, *P* = 0.008 (CIs: −1.0, −0.49). ISEQ scores were also unrelated to motor thresholds, *r*_s_ = .05, *P* = 0.93 (CIs: −0.97, 0.85), although the difference between the correlations was nonsignificant (median difference: 0.83; CIs: −0.19, 1.80).

Cumulatively, these results demonstrate that phosphene thresholds are selectively lower in synesthetes and also vary as a function of individual differences in the visuospatial phenomenology of synesthesia. Specifically, those synesthetes who experience color photisms as spatially colocalized with inducing graphemes (projectors) exhibit greater visual, but not motor, cortical excitability than those who experience color photisms as visual images (associators), with both subtypes displaying greater cortical excitability than controls. This effect was also replicated when visuospatial phenomenology was treated as a continuous, rather than categorical, variable.

### Synesthetes do not Exhibit Atypical Neurochemical Profiles but Visual Cortex Glutamate Concentrations Predict Synesthesia Phenomenology

Our final analyses investigated whether atypical phosphene thresholds in synesthetes were driven by atypical GABA or glutamate concentrations in visual cortex (Fig. 3*B*,*C*). A 2 × 2 mixed-model ANOVA on glutamate concentrations revealed a main effect of Region, *F*_1,18_ = 54.28, *P* < 0.001, *η*_p_^2^ = 0.75 (CIs: 0.47, 0.84), reflecting greater glutamate concentrations in visual than motor cortex, but no Group difference, *F*_1,18_ = 2.43, *P* = 0.14, *η*_p_^2^ = 0.12 (CIs: 0.00, 0.39), nor an interaction, *F*_1,18_ = 0.15, *P* = 0.70, *η*_p_^2^ = 0.01 (CIs: 0.00, 0.21). The Region effect became nonsignificant when controlling for gray and white matter percentages in each voxel, *F* < 2.8. There were no effects of Region, *F*_1,18_ = 1.02, *P* = 0.33, *η*_p_^2^ = 0.05 (CIs: 0.00, 0.31) or Group, *F*_1,18_ = 0.12, *P* = 0.78, *η*_p_^2^ = 0.01 (CIs: 0.00, 0.20), nor an interaction, *F*_1,18_ = 0.26, *P* = 0.62, *η*_p_^2^ = 0.01 (CIs: 0.00, 0.23), on GABA concentrations. This suggests that synesthetes do not differ from controls in glutamate or GABA concentrations.

We next investigated whether glutamate or GABA concentrations differed as a function of synesthesia phenomenology (Fig. 3*B*,*C*). Mixed-model ANOVAs contrasting controls and the 2 synesthesia subtypes on GABA and glutamate concentrations replicated the main effect of Region on glutamate concentrations, *F*_1,17_ = 46.44, *P* < 0.001, *η*_p_^2^ = 0.73 (CIs: 0.43, 0.83), but no effects of Group, *F*_2,17_ = 1.20, *P* = 0.33, *η*_p_^2^ = 0.12 (CIs: 0.00, 0.36), nor an interaction, *F*_2,17_ = 1.43, *P* = 0.27, *η*_p_^2^ = 0.14 (CIs: 0.00, 0.38). The Region effect again became nonsignificant when controlling for gray and white matter percentages within each voxel, *F* < 2.2. There were no main effects of Region, *F*_1,17_ = 3.75, *P* = 0.07, *η*_p_^2^ = 0.18 (CIs: 0.00, 0.45), Group, *F*_2,17_ = 0.58, *P* = 0.57, *η*_p_^2^ = 0.06 (CIs: 0.00, 0.28), nor an interaction, *F*_2,17_ = 2.82, *P* = 0.09, *η*_p_^2^ = 0.25 (CIs: 0.00, 0.48) on GABA concentrations.

The ISEQ associator–projector provides a more fine-grained measure of individual differences among synesthetes and so we next explored associations between this measure and neurochemical concentrations. ISEQ scores (higher values indicate greater projector-type phenomenology) positively correlated with visual cortex glutamate concentrations, *P* = 0.03 (Fig. 3*D*), but not GABA concentrations, *P* = 0.18 (Fig. 3*E*). The former correlation was statistically independent of visual cortex GABA concentrations, *r*_ps_ = 0.73, *P* = 0.04 (CIs: 0.06, 0.98), but the difference between the ISEQ correlations with glutamate and GABA were not different (median difference: 0.17, CIs: −0.52, 1.20). In contrast, ISEQ scores were unrelated to motor cortex glutamate concentrations, *P* = 0.15 (Fig. 3*F*). The magnitude of this correlation was significantly different from that between visual cortex glutamate concentrations and ISEQ scores (median difference: 1.22, CIs: 0.43, 1.84), and the correlation between ISEQ scores and visual cortex glutamate concentrations remained suggestive when controlling for motor cortex glutamate concentrations, *r*_ps_ = 0.68, *P* = 0.065 (CIs: −0.19, 0.98). Finally, ISEQ scores were unrelated to motor cortex GABA concentrations, *r*_s_ = −0.43, *P* = 0.25 (CIs: −0.95, 0.47). Taken together, these results indicate that controls and synesthetes do not differ in basal glutamate or GABA concentrations in motor or visual cortex but suggest that individual differences in color photism visuospatial phenomenology among synesthetes are selectively associated with glutamate concentrations in visual cortex, with projector phenomenology being associated with elevated glutamate concentrations.

## Discussion

This study investigated the neurochemical basis of phosphene thresholds and their relation to individual differences in visuospatial awareness. We observed that phosphene thresholds selectively predicted local basal concentrations of glutamate in primary visual cortex. Lower phosphene thresholds, typically interpreted to reflect elevated cortical excitability, were associated with higher glutamate concentrations. This relationship was highly specific and strong in magnitude, with glutamate concentrations accounting for approximately 50% of the variance in phosphene thresholds. We also found that phosphene thresholds covaried with the visuospatial phenomenology of synesthesia with projectors exhibiting lower phosphene thresholds than associators, with both displaying lower thresholds than controls. Glutamate concentrations in primary visual cortex were also associated with individual differences in the phenomenology of synesthesia but did not differ between controls and synesthetes. These results provide evidence for the neural substrate underlying variability in phosphene thresholds and suggest that phosphene perception and glutamate concentrations relate to individual differences in visuospatial awareness.

The observed relationship between phosphene thresholds and visual cortex glutamate concentrations suggests that interindividual differences in phosphene perception are strongly driven by basal glutamatergic excitation in primary visual cortex. This result provides a crucial validation of the widespread usage of phosphene thresholds as a measure of visual cortex excitability (Cowey and Walsh 2000; Boroojerdi et al. 2001; Stewart et al. 2001; Antal et al. 2003; Bestmann et al. 2007) and parallels similar results in motor cortex (Stagg et al. 2011). This relationship was insensitive to a range of possible confounding variables and displayed neurochemical, topographic, and threshold specificity, although the results are limited because we did not use MRI to localize TMS stimulation sites. Crucially, the association between visual cortex glutamate concentrations and phosphene thresholds was observed independently in both controls and synesthetes, with correlations of comparable magnitude, suggesting that this relationship may generalize to both the general population and subpopulations characterized by atypical visual processing. Previous research has demonstrated that in typical populations phosphene thresholds are inversely related to resting state occipital *α*-band power (∼8–14 Hz) (Romei, Rihs, et al. 2008) and intra-individual variability in phosphene perception is driven in part by endogenous fluctuations in prestimulus *α*-band power and phase (Romei, Brodbeck, et al. 2008; Dugué et al. 2011; Romei et al. 2012). Accordingly, one potentially fruitful avenue for further research will be to investigate the relations between occipital *α*-band and γ-band (Terhune et al. 2015) power and glutamate concentrations and their unique and overlapping contributions to individual differences in phosphene perception.

We did not observe a comparable relationship between motor thresholds and motor cortex glutamate concentrations. A previous study demonstrated such a relationship (Stagg et al. 2011) but used motor-evoked potential input–output curves to determine motor thresholds, rather than the observation of movement method (Varnava et al. 2011). We used the latter because it relies on subjective judgments and thus provides a superior control for phosphene thresholds than a method based on motor-evoked potentials. Although the observation of movement method is highly reliable (Varnava et al. 2011), the present results suggest that motor thresholds derived with this method are unrelated to motor cortex glutamate levels. Further research is required to directly contrast motor-evoked potential input–output curve and observation of movement methods in the prediction of motor cortex glutamate concentrations.

Our results also provide further evidence regarding the functional relationship between phosphene thresholds and visual cognition. As independently predicted (Brogaard 2014), projector synesthetes displayed greater cortical excitability in primary visual cortex than associator synesthetes, both of whom exhibited hyperexcitability relative to controls, thus replicating our previous work (Terhune et al. 2011). These differences between synesthesia subtypes also parallel our recent finding that projectors display lower phosphene thresholds than controls and associators selectively with 40 Hz transcranial alternating current stimulation (Terhune et al. 2015). Although they should be interpreted with caution given the sample sizes, these differences expand upon research showing that spatial attention transiently enhances excitability in primary visual cortex (Bestmann et al. 2007) by demonstrating that phosphene perception varies with interindividual variability in visuospatial awareness. Indeed, we replicated the relation between visuospatial phenomenology and phosphene thresholds when we treated associator–projector subtype as a continuous measure (Skelton et al. 2009), indicating that this relationship holds with a more fine-grained measure of individual differences in this population. Such an approach will be valuable in elucidating the neural basis of synesthesia phenomenology (Rouw and Scholte 2010; van Leeuwen et al. 2011). We further provide preliminary evidence linking associator–projector phenomenology and visual cortex glutamate concentrations: Synesthetes experiencing greater projector phenomenology exhibited selectively higher concentrations of glutamate in visual cortex. This effect displayed neurochemical and topographic specificity and was not observed with visual cortex GABA or motor cortex glutamate concentrations. Given the results of Bestmann et al. (2007), one interpretation of the observed difference is that projectors' perception of color photisms as spatially proximal to inducing graphemes enhances cortical excitability over time, with concomitant attenuation of phosphene thresholds and enhancement of glutamate concentrations, possibly reflecting visual cortex plasticity (see, e.g., Boroojerdi et al. 2001). Alternatively, visual cortex hyperexcitability in projectors may contribute to the evocation of a spatially localized reference frame. Irrespective of the causal direction of this relationship, the observed difference across synesthesia subtypes is consistent with the proposal that associators and projectors primarily differ in the spatial reference frame evoked upon presentation of an inducing grapheme (Ward et al. 2007).

The observed relationships pertaining to synesthesia are also consistent with previous research on visual processing and attention in this population. In particular, the observed difference between subtypes parallels the finding that projectors' synesthetic associations are more strongly influenced by low-level visual properties of the inducing grapheme (Brang et al. 2011). Projectors also exhibit larger synesthetic Stroop effects and have quicker conscious detection of graphemes among distractors, than associators (Dixon et al. 2004; Palmeri et al. 2002). These effects may transpire because grapheme-color binding occurs at an earlier visual processing stage in projectors (Van Leeuwen et al. 2011). More broadly, previous research on the electrophysiological and cognitive correlates of phosphene perception (Sparing et al. 2002; Hoffken et al. 2013) suggests that visual cortex hyperexcitability among synesthetes is plausibly associated with enhanced visual processing in this population (Barnett et al. 2008). This is consistent with research demonstrating a reduced ability to experience phosphenes in individuals with impaired vision (Gothe et al. 2002), which further links cortical excitability and visual perception. Associations between reduced occipital *α*-band power and *α* phase dynamics and phosphene perception (Romei, Brodbeck*,* et al. 2008; Dugué et al. 2011; Romei et al. 2012) suggest that projectors will display lower occipital *α*-band power than associators and controls. Projectors that we have studied have reported variability in the perceived visuospatial position of color photisms although this has not been systematically studied to our knowledge. This variability may depend on the prestimulus phase of *α* oscillations (see, e.g., Dugué et al. 2011). More broadly, lower prestimulus *α*-band power is associated with superior visual perception (Hanslmayr et al. 2007), depending on the task (Lange et al. 2013), and thus may relate to superior visual processing among synaesthetes (Barnett et al. 2008; Banissy et al. 2013; Terhune, Song, et al. 2014), which might translate to cognitive domains relying on vision such as working memory (Terhune, Wudarczyk, et al. 2013). Whether the current results suggest superior visual perception among projectors in particular merits attention. Color synesthetes display superior color processing, but impaired motion processing (Banissy et al. 2013), supporting the view that the benefits conferred by synesthesia have neurological costs (Cohen Kadosh et al. 2012). Coupled with the current results, this leads to the prediction that color synesthetes will display elevated (or normal) thresholds for moving phosphenes during MT/V5 stimulation (e.g., Pascual-Leone and Walsh 2001).

The current results provide an important qualification regarding the relationship between phosphene thresholds and glutamate concentrations in primary visual cortex. In particular, although synesthetes displayed lower phosphene thresholds than controls, they did not exhibit correspondingly lower glutamate concentrations than controls as might be expected given the correlation between phosphene thresholds and glutamate concentrations. This discrepancy closely parallels results pointing to a lack of abnormal glutamate levels in individuals with migraine with aura (Reyngoudt et al. 2012), despite reduced phosphene thresholds in this population (Brigo, Storti, et al. 2013), and is in accordance with research suggesting an association between synesthesia and migraine (Podoll and Robinson 2002; Alstadhaug and Benjaminsen 2010).

One reason for the lack of atypical glutamate concentrations in synesthesia may be that elevated glutamate contributes to the emergence, and expression, of synesthesia at an early developmental stage, as part of broad differences in temporal, parietal, and occipital cortices (Rouw and Scholte 2010; van Leeuwen et al. 2011). However, as synesthetes age, persistent concurrent activation of downstream regions, including the fusiform gyrus, V4, and parietal cortex, may lead to insufficient pruning during infancy and thus increased connectivity, which may then play a central role in the maintenance of synesthesia and its phenomenology (van Leeuwen et al. 2011), with normalization of glutamate (Terhune et al. 2011). Alternatively, elevated cortical excitability in this population may be driven by atypical glutamate receptor activity, rather than increased concentration. Glutamate plays an important role in cone signaling within the visual system, transmitting high-frequency signals to postsynaptic bipolar cells (Jackman et al. 2009). A small amount of glutamate spillover between cones occurs, due to saturation of receptor mechanisms at cone terminals (Szmajda and Devries 2011), resulting in a spread of excitation to neighboring cones, thereby facilitating glutamatergic crosstalk. Dysfunctional glutamate receptor activity in individuals with synesthesia may result in greater spread of glutamate beyond its intended postsynaptic target, and thus lower phosphene thresholds, while maintaining normal glutamate concentrations. Further research is required to assess the viability of these competing explanations. The first hypothesis could be tested by measuring glutamate concentrations in synesthetic infants and children, who would be expected to have elevated glutamate levels selectively in primary visual cortex. The second, on the other hand, will be more difficult to interrogate because it will require the development of an animal model of synesthesia and only preliminary research has been done toward this end (Neely et al. 2010; Brang and Ramachandran 2011; Terhune, Rothen, et al. 2013).

In conclusion, our findings demonstrate that TMS phosphene thresholds are strongly, negatively related to local concentrations of basal glutamate in primary visual cortex and both phosphene thresholds and visual cortex glutamate concentrations covary with the visuospatial phenomenology of grapheme-color synesthesia. These findings suggest that individual differences in phosphene perception are driven by variability in local basal glutamate levels in primary visual cortex and provide an important validation for the widespread use of phosphene thresholds as a measure of cortical excitability. They further suggest that phosphene perception may relate to individual differences in visuospatial awareness.

## Authors’ Contributions

Devin B. Terhune, Alan Cowey, and Roi Cohen Kadosh conceived and designed the study. Devin B. Terhune and Elizabeth Murray collected the data. Devin B. Terhune, Elizabeth Murray, Jamie Near, and Charlotte J. Stagg analyzed the data. Devin B. Terhune wrote the manuscript, and Elizabeth Murray, Jamie Near, Charlotte J. Stagg, and Roi Cohen Kadosh provided feedback.

## Funding

This research was supported by the Cogito Foundation to D.B.T. He is also supported by a Marie Skłodowska-Curie Intra-European Fellowship within the 7th European Community Framework Programme. R.C.K. is supported by the Wellcome Trust (WT88378). Funding to pay the Open Access publication charges for this article was provided by Wellcome Trust (WT88378).
